# Estimation error in speed of sound caused by rotation of measured cross-section from short-axis plane of blood vessels: a preliminary study

**DOI:** 10.1007/s10396-023-01383-y

**Published:** 2023-11-30

**Authors:** Shohei Mori, Keiji Onoda, Mototaka Arakawa, Hiroshi Kanai

**Affiliations:** 1 Graduate School of Engineering, Tohoku University, 6-6-05 Aoba, Aramaki, Aoba, Sendai, Miyagi 980-8579 Japan; 2 Graduate School of Biomedical Engineering, Tohoku University, 6-6-05 Aoba, Aramaki, Aoba, Sendai, Miyagi 980-8579 Japan

**Keywords:** Ultrasound imaging, Speed of sound, Liver, Blood vessels, Element data, Delay time

## Abstract

**Purpose:**

Estimating the speed of sound (SoS) in ultrasound propagation media is important for improving the quality of B-mode images and for quantitative tissue characterization. We have been studying a method for estimating the SoS by measuring the reception time distribution of waves scattered from a scatterer at the elements in a probe. Previously, the measurement cross section was assumed to be perpendicular to the long axis of the blood vessel. In this study, we experimentally investigated the relationship between rotation angle $\theta$ of the probe relative to the short-axis plane of the blood vessel and the estimated SoS, $\widehat{c}$.

**Methods:**

Water tank and phantom experiments were conducted to investigate the characteristics of $\widehat{c}$ and element signals when the probe was rotated.

**Results:**

The received signal powers at the elements around both edges greatly decreased as $\theta$ increased. We introduced a parameter representing the decrease in power, ${P}_{\mathrm{dec}}$, in the received signal at the elements at both edges relative to the center element. $\widehat{c}$ was estimated to be larger as $\theta$ increased, especially for $\theta \ge 30^\circ$. ${P}_{\mathrm{dec}}$ also increased as $\theta$ increased. An approximately proportional relationship existed between the errors in $\widehat{c}$ and ${P}_{\mathrm{dec}}$.

**Conclusion:**

Based on these results, we can distinguish between the presence and the absence of SoS misestimations using the difference in power among the elements in the received signal. In the absence of misestimation, we can obtain the true SoS, even if the target has a non-negligible size, by applying our previously proposed methods.

## Introduction

Medical ultrasound imaging is useful for observing various organs because of its noninvasiveness and real-time performance. The formation of a B-mode image from the received element signals requires information on the speed of sound (SoS) in the propagation medium. The image quality degrades when an image is formed at an assumed SoS that differs from the actual SoS. Therefore, several studies have been conducted to estimate the SoS in propagation media [1–9]. Determination of the SoS in tissues can also be used to identify diseases. For example, the SoS in the liver decreases with an increase in fat concentration [10]. Therefore, methods for estimating the SoS in the liver have been studied to quantitatively diagnose fatty liver [11–13].

We proposed a method for estimating the SoS in propagation media by measuring the reception time distribution of waves scattered from a scatterer at elements in a linear array probe [4]. To accurately estimate the SoS using this method, the reception time distribution must be measured accurately. For this purpose, the use of signals with a high signal-to-noise (SNR) ratio is preferable. The liver contains many blood vessels, and the signals received from these vessels have a higher intensity than the scattered waves from the liver parenchyma. We have studied a method to estimate the average SoS of an ultrasound propagation medium between the probe and blood vessels in the liver using signals from blood vessels with high signal intensity.

Our group developed a method to estimate the SoS using the reception time of a scattered wave from a point scatterer at each element [4]. However, the assumption of a point scatterer is not applicable to scatterers larger than the ultrasound wavelength, such as blood vessels. Therefore, we verified the effect of the size of the scatterers in the SoS estimation method assuming a point scatterer. We observed that SoS $\widehat{c}$ was overestimated (positive bias error) when the scatterers had non-negligible diameters [14]. Therefore, we proposed a method for correcting the overestimated $\widehat{c}$ using the apparent size and depth of the scatterer in the B-mode image [15]. This method can estimate the average SoS in the ultrasound propagation region using signals from scatterers of a finite size, such as blood vessels. To apply our previous studies [14, 15] to SoS estimation using signals from a blood vessel, the measurement cross section must be perpendicular to the long axis of the blood vessel. However, this assumption does not always hold because of the complex directions of the blood vessels in the liver [16, 17]. In this study, the cross-section perpendicular to the long axis of the blood vessel was defined as the short-axis plane.

**Fig. 1:**
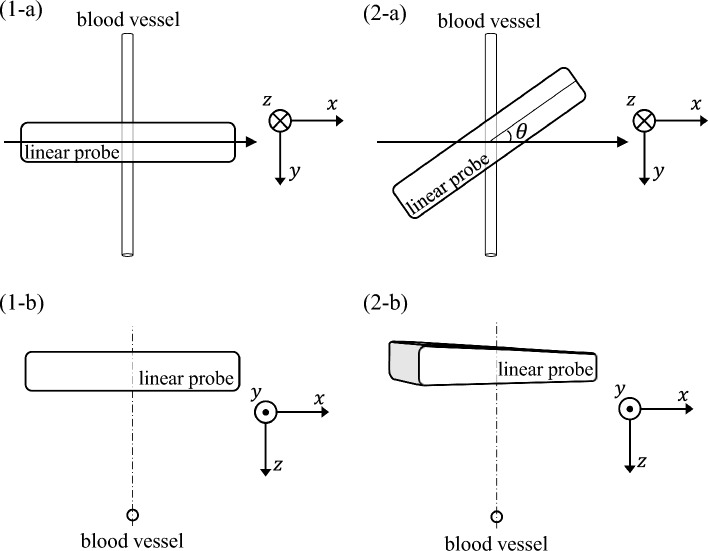
Schematic of measurement of a blood vessel using an ultrasound probe. (1-**a**, **b**) Ultrasound probe measuring the short-axis plane of a blood vessel and (2-**a**, **b**) when the probe is $\theta$-rotated around the *z*-axis relative to the short-axis plane of a blood vessel

Figure 1 shows a schematic of the blood vessel measurement using an ultrasound probe. Figure 1(1) shows the probe measuring the short-axis plane of the blood vessel, and Fig. 1(2) shows the probe rotating at an angle $\theta$ around the *z*-axis relative to the short-axis plane of the blood vessel. In this paper, we experimentally investigated the relationship between probe rotation angle $\theta$ relative to the short-axis plane of the blood vessel and the estimated $\widehat{c}$, and observed that $\widehat{c}$ was overestimated by the SoS estimation method assuming a point scatterer when $\theta$ was large. Furthermore, since $\theta$ is unknown in actual in vivo measurements, we propose a method to determine whether the method described in Ref. [4] could be applied, based on the characteristics of the received signal measured at each element and without using the information of $\theta$.

## Methods

### Speed-of-sound estimation assuming an ideal point scatterer as a target [4]

In our previous study [4], we proposed a method for estimating the SoS distribution when the medium is not homogeneous. In this paper, we describe an SoS estimation method using a linear array probe for a homogeneous medium because the present study is a basic investigation.

The target of the ultrasound irradiation is assumed to be an ideal point scatterer at depth $d$ immediately below the center element of the linear array probe. The position of the central element is defined as ${x}_{k}=0$. A wave scattered from the point scatterer is received at each element in the probe. Let $c$ be the average SoS in the ultrasound propagation medium, ${x}_{k}$ be the element position of element number $k$, and ${T}_{0}({x}_{k})$ be the theoretical value of the return propagation time of the scattered wave from the point scatterer at element $k$. ${T}_{0}({x}_{k})$ can be expressed by Eq. (1).

1 $${T_{0} \left( {x_{k} } \right) = \frac{{\sqrt {x_{k}^{2} + d^{2} } }}{c}}$$

Taking the square of Eq. (1), the right-hand side represents a linear expression for ${x}_{k}^{2}$.

2 $$\begin{array}{c}{\left({T}_{0}\left({x}_{k}\right)\right)}^{2}=\frac{1}{{c}^{2}}{x}_{k}^{2}+\frac{{d}^{2}}{{c}^{2}}=a{x}_{k}^{2}+b.\end{array}$$

Squared values $\left\{{\left({T}_{0}\left({x}_{k}\right)\right)}^{2}\right\}$ of theoretical values ${\{T}_{0}({x}_{k})\}$ for the return path propagation times are matched with the squared values of measured values $\{t\left({x}_{k}\right)\}$ of the return path propagation times of the scattered wave at element $k$. Coefficients $a$ and $b$ in Eq. (2) are determined using the least-squares method. Furthermore, by comparing the coefficients of the second and third equations in Eq. (2), average $c$ from the probe to the scatterer and depth of the scatterer, $d$, can be simultaneously estimated using Eqs. (3) and (4), respectively.

3 $$\widehat{c}=\sqrt{\frac{1}{a}} ,$$

4 $$\widehat{d}=\sqrt{\frac{b}{a}} .$$

### Effects of using signals from blood vessels in the speed-of-sound estimation method

An ideal point scatterer is assumed in the aforementioned SoS estimation method. However, when the scatterer has a finite size, the irradiated ultrasonic wave is scattered at each position on the surface of the scatterer, and the arrival time of the scattered wave to each element is earlier than that of the ideal point scatterer, except for the center element. Thus, $a$ in Eq. (2), decreases, and $\widehat{c}$ using Eq. (3) is overestimated [14]. When the propagation path of each ultrasound wave is only on the short-axis plane of the blood vessel, the positive bias error in the estimated SoS caused by the size of the scatterer can be corrected based on the geometric relationship between the apparent size and depth of the scatterer measured on the B-mode image and the reception time at each element [15].

Whereas previous studies [14, 15] assumed that the short-axis plane of a cylindrical target was measured as shown in Fig. 1(1), this study investigated the condition in which the probe is $\theta$-rotated around the *z*-axis relative to the short-axis plane of the vessel [Fig. 1(2)]. The beam width along the elevational direction of the probe is several millimeters, and the blood vessels have a cylindrical shape. Therefore, when the probe is $\theta$-rotated relative to the short-axis plane of the vessel, the irradiated ultrasonic wave is scattered on the uppermost surface of the vessel cylinder across the imaging plane of the probe, and the reception time of the scattered waves received at each element becomes earlier, except for the center element, than that when $\theta =0^\circ$, causing a positive bias error in the estimated $\widehat{c}$ in Eq. (3).

This change in the reception time of the scattered waves may be altered by the transmitted beam condition and depth of the target cylinder. However, if this relationship does not depend on the target tissue characteristics, such as SoS and attenuation of the propagation medium and SoS of the target blood vessel, we can preliminarily measure and understand the relationship between rotation angle $\theta$ and estimated SoS $\widehat{c}$ for each machine setting and target depth. Thus, in this preliminary study, we examined a single condition of the transmitted beam and target depth. Meanwhile, the dependence of the relationship between $\theta$ and $\widehat{c}$ on the target tissue characteristics must be confirmed. Therefore, in this study, we conducted two experiments using a silicone tube in a water tank and nylon wires in a phantom, with different acoustic properties (SoS and attenuation of propagation media and SoS of target cylinders).

### Water tank experiment

First, we conducted a simple experiment in which a silicone rubber tube was placed in a water tank. The probe was rotated by $\theta$ relative to the short-axis plane of the silicone tube [Fig. 1(2)], and the characteristics of estimated $\widehat{c}$ and element signals changed by $\theta$ were investigated. The diameters of the target blood vessels in the human liver range from a few hundred micrometers to a few millimeters [16]. However, to distinguish the effect of probe rotation angle $\theta$ from that of the scatterer size described in previous studies [14, 15], we used a thin tube with inner and external diameters of 100 and 200 μm, respectively. The silicone tube was placed 30 mm from the probe surface. The true SoS in the water was obtained by measuring the water temperature [18].

### Phantom experiment

**Fig. 2:**
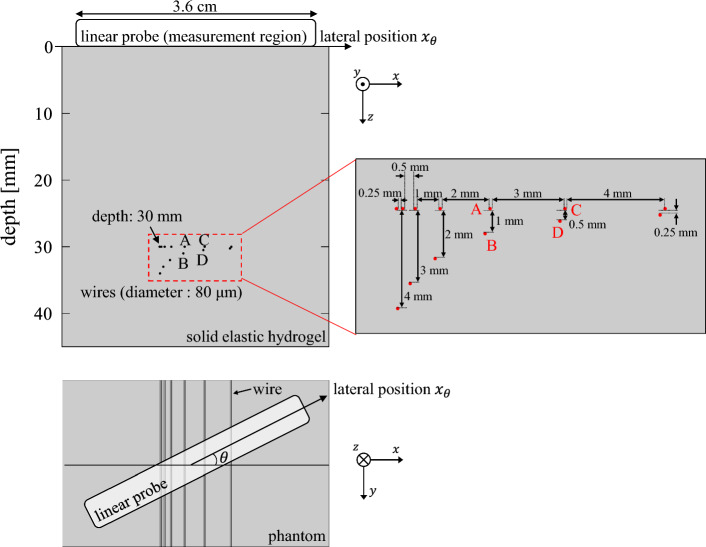
Schematic of the phantom experiment

A phantom experiment was conducted to simulate the liver condition in which the target cylinder (blood vessel) was surrounded by a weak scattering source (such as liver parenchyma) and to examine the conditions of different acoustic properties from those in the water tank experiment. We used a nylon wire with a diameter of 80 μm, which is shorter than the wavelength, as the target signal for SoS estimation to distinguish the effect of probe rotation angle $\theta$ from that of the scatterer size described in previous studies [14, 15]. A general-purpose ultrasound phantom (Model 054GS; CIRS, USA) was used. A region with 13 wires aligned at different distances was measured, as shown in Fig. 2. The wires were surrounded by a hydrogel with a nominal SoS of $1540\pm 10$ m/s.

### Acoustic properties of ultrasound propagation media and target cylinders in water tank and phantom experiments

The SoS and attenuation coefficient in the propagation media in the water tank (water [18, 19]) and phantom (hydrogel) experiments and the SoS in the target cylinders (silicone tube [20] and nylon wire [21]) are summarized in Table 1. As shown in Table 1, the acoustic properties in the water tank and phantom experiments differed.

**Table 1:** Acoustic properties in water tank and phantom experiments

	Propagation medium		Speed of sound in target cylinder [m/s]
	Speed of sound [m/s]	Attenuation coefficient [dB/cm/MHz]	
Water tank experiment	1486–1491 [18] (water)	0.0021 (at 20 $^\circ{\rm C}$) [19] (water)	960 [20] (silicone rubber)
Phantom experiment	1540 $\pm$ 10 (nominal value) (hydrogel)	0.7 (nominal value) (hydrogel)	2530 [21] (nylon)

### Ultrasonic measurement conditions

An ultrasound diagnostic apparatus (Prosound SSD-α10; Hitachi Aloka, Japan) with a linear array probe (UST-5412; Hitachi Aloka, Japan) was used for data acquisition. The element pitch was 0.20 mm, transmitting frequency was 7.5 MHz, and sampling frequency was 40 MHz. A total of 95 elements were used for the transmission and reception of the ultrasound beam. The focal depth was set at 30 mm. For each target cylinder, the received element signals acquired using the ultrasonic beam transmitted from the element group centered on the element above the target cylinder were analyzed to estimate the SoS. The possible maximum deviation of lateral positions between the center of the beam and that of the target cylinder is 0.10 mm (half the distance between adjacent elements). The probe was rotated by $\theta$ around the *z*-axis, as shown in Fig. 2, and data were acquired at *θ* =0°, 10°, 20°, 30°, and $60^\circ$ to examine a broader range of rotation angle $\theta$.

## Results

### Water tank experiment

**Fig. 3:**
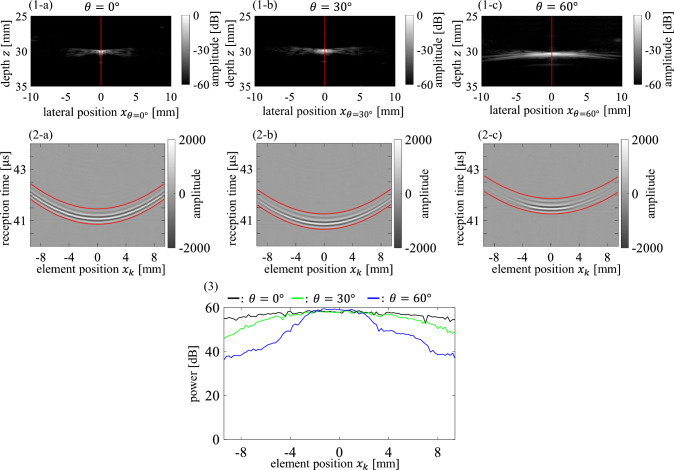
Measurement results when the probe was rotated $\theta =0^\circ$, $30^\circ$, and $60^\circ$ relative to the silicone tube arranged in a water tank. (1) B-mode images of the silicone tube for (1-**a**) $\theta =0^\circ$, (1-**b**) $\theta =30^\circ$, and (1-**c**) $\theta =60^\circ$. (2) Received element signals from the silicone tube for (2-**a**) $\theta =0^\circ$, (2-**b**) $\theta =30^\circ$, and (2-**c**) $\theta =60^\circ$. (3) Average power of the received signal at each element in the window (red lines) in (2)

The measurement results for the silicone tube in water at *θ* = 0°, 30°, and $60^\circ$ are shown in Fig. 3. Figure 3(1) shows the B-mode images. The observed shapes of the silicone tube on the B-mode images extended along the lateral direction as $\theta$ increased. The ultrasonic beam had a width in the elevational direction, and the silicone tube had a cylindrical shape. Therefore, the observed shapes on the B-mode images were considered to extend along the lateral direction because the signal from the silicone tube was received on the beams near the center of the tube, similar to that on the center beam, for $\theta =30^\circ$ and $60^\circ$.

Figure 3(2) shows the received element signals before the formation of the ultrasonic beam. The signals received from the silicone tube had smaller amplitudes at the elements around both edges as probe rotation angle $\theta$ increased.

Figure 3(3) shows the average power of the received element signals in a 0.6-µs wide window between the two red lines shown in Fig. 3(2), calculated for each element. The window for calculating the power of the received signals was set along the approximated time distribution obtained using Eqs. (1)–(4). As shown in Fig. 3(3), the signal powers at the elements around both edges greatly decreased as $\theta$ increased.

### Phantom experiment

**Fig. 4:**
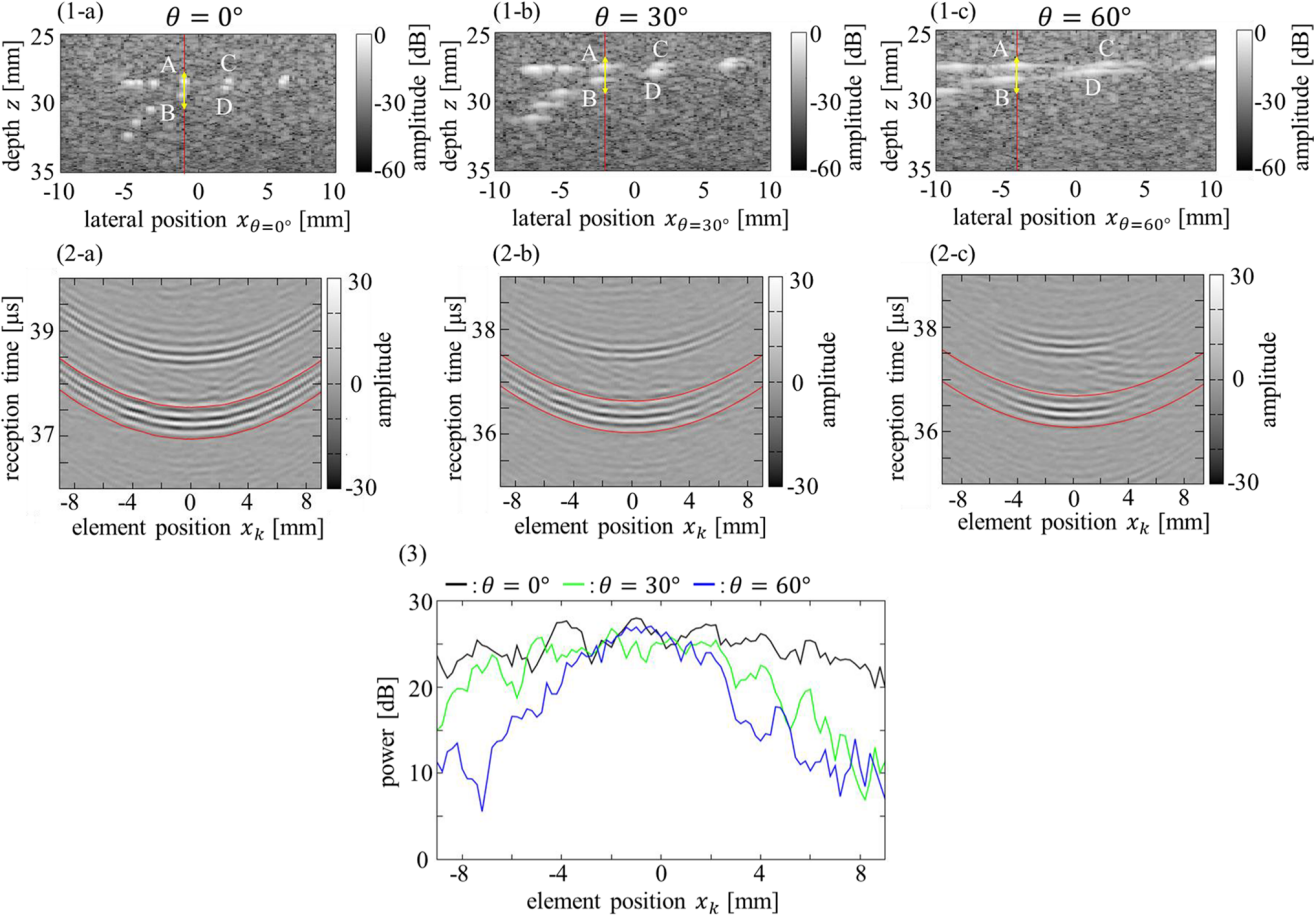
Measurement results when the probe was rotated $\theta =0^\circ$, $30^\circ$, and $60^\circ$ relative to the phantom. (1) B-mode images of the phantom for (1-**a**) $\theta =0^\circ$, (1-**b**) $\theta =30^\circ$, and (1-**c**) $\theta =60^\circ$. (2) Received element signals from scatterer A for (2-**a**) $\theta =0^\circ$, (2-**b**) $\theta =30^\circ$, and (2-**c**) $\theta =60^\circ$. (3) Average power of the received signal at each element in the window (red lines) in (2)

The measurement results for the phantom at $\theta =0^\circ$, $30^\circ$, and $60^\circ$ are shown in Fig. 4. Figure 4(1) shows B-mode images. Figure 4(2) shows the received element signals before the formation of the ultrasonic beam near wire A at each angle $\theta$, where the ranges of the signals are indicated by the arrows in Fig. 4(1). Similar to the results of the water tank experiment, the observed shapes of the nylon wire on the B-mode images extended along the lateral direction and the element signals showed smaller amplitudes at the elements around both edges as $\theta$ increased.

Figure 4(3) shows the average power of the received signals in a 0.6-µs wide window between the two red lines shown in Fig. 4(2), calculated for each element. As the detection of signals from the nylon wire at the edge-side elements was difficult owing to the decrease in the signal amplitude, the parabolic approximation for SoS estimation using Eq. (2) was applied only to the elements whose peak signals could be detected, and the window for calculating the power of the received signals was set along the approximated time distribution obtained using Eqs. (1)–(4).

The power of the received signals from the nylon wire was lower than that from the silicone tube in water (Fig. 3) because of the difference in attenuation between the hydrogel and water (approximately − 30 dB difference when the 7.5 MHz frequency of the ultrasound wave propagated a total round-trip distance of 60 mm). However, similar to the water tank experiment, the decrease in power around the edge of elements due to the increase in $\theta$ was observed for the phantom experiment, as shown in Fig. 4(2) and (3).

### Relationship between the decrease in power and the SoS estimation error

To quantify the decrease in the received power at the elements at both edges ($k=\pm 47$) relative to the center element ($k=0$), we introduced a parameter representing the decrease in power, ${P}_{\mathrm{dec}}$, defined by the following equation:

5 $$\begin{array}{c}{P}_{\mathrm{dec}}=\frac{{P}_{{x}_{k=-47}}+{P}_{{x}_{k=47}}}{2}-{P}_{{x}_{k=0}}, \left[\mathrm{dB}\right]\end{array}$$

where ${P}_{{x}_{k}}$ denotes the average power of the signals in the window, calculated for the element at position ${x}_{k}$.

**Fig. 5:**
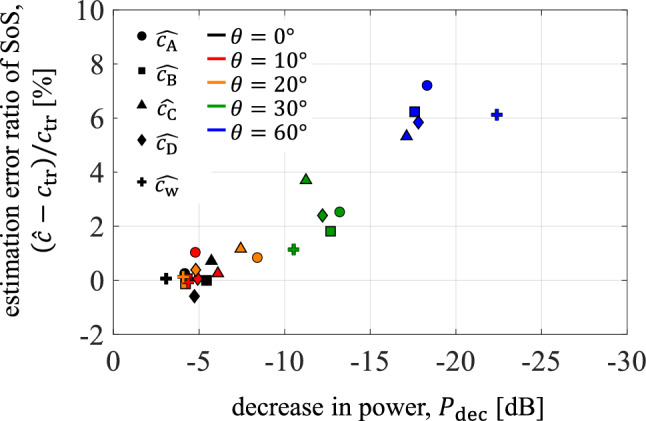
Relationship between the decrease in power, ${P}_{\mathrm{dec}}$, at each angle $\theta$ and the speed-of-sound (SoS) estimation error ratio, $(\widehat{c}-{c}_{\mathrm{tr}})/{c}_{\mathrm{tr}}$, where ${c}_{\mathrm{tr}}$ is the true SoS. $\widehat{{c}_{\mathrm{A}}}$, $\widehat{{c}_{\mathrm{B}}}$, $\widehat{{c}_{\mathrm{C}}}$, and $\widehat{{c}_{\mathrm{D}}}$: SoS in phantom (hydrogel) estimated using the signals received from nylon wires A, B, C, and D, respectively. $\widehat{{c}_{\mathrm{w}}}$: SoS in water estimated using signals from silicone tube

The $\widehat{c}$ values were estimated by applying the SoS estimation method in [4] to the signals received from the silicone tube in water and nylon wires A, B, C, and D in the phantom. The relationships between the ${P}_{\mathrm{dec}}$ values calculated using Eq. (5) and the SoS estimation error ratios, $\left(\widehat{c}-{c}_{\mathrm{tr}}\right)/{c}_{\mathrm{tr}}$, where ${c}_{\mathrm{tr}}$ is the true SoS, are shown in Fig. 5. The results of both the water tank and phantom experiments are plotted in Fig. 5 (the results of the water tank experiment are plotted by cross markers).

As ${P}_{\mathrm{dec}}$ decreased, the SoS estimation error increased in both water tank and phantom experiments. This result showed that even if $\theta$ is unknown, the thresholding process for ${P}_{\mathrm{dec}}$ can estimate and eliminate the conditions under which $\theta$ is large and the SoS is misestimated.

For $\theta \le 30^\circ$, the water tank and the phantom experiments showed a similar relationship between ${P}_{\mathrm{dec}}$ and SoS estimation error ratio. This result indicates that the relationship between the ${P}_{\mathrm{dec}}$ and the SoS estimation error ratio is independent of or less dependent on the acoustic properties of the propagation medium and target cylinders.

For $\theta =60^\circ$, however, ${P}_{\mathrm{dec}}$ in the phantom experiment was higher than that in the water tank experiment, whereas the SoS estimation error ratio showed similar values. This difference in ${P}_{\mathrm{dec}}$ might be caused by the lower SNR in the phantom experiment. In the phantom, the nylon wires were surrounded by hydrogel; therefore, the scattered signals from the nylon wires were affected by the interference of the weak scattered signals from the hydrogel. The averaged power of the scattered signals from the hydrogel was 7 dB in both the regions at depths of 25.0–25.5 mm and 35.0–35.5 mm, averaged for all 95 elements. Because the transmitted focus depth was 30 mm, the powers of the scattered signals from the hydrogel at depths of nylon wires were considered to be higher than 7 dB. Therefore, the powers of the scattered signals from the hydrogel were on the same order as those from the target wires (approximately 10 dB) at both edge elements for $\theta =60^\circ$, as shown in Fig. 4(3). Thus, the decrease in the power of the wire signals around the edge elements was considered to be suppressed compared with the noiseless condition in the water tank experiment.

In Fig. 5, the results of the phantom experiment deviate slightly for wires A, B, C, and D. This may be attributed to the interference of signals from the surrounding scattering sources (hydrogel) and the low SNR due to attenuation in the propagation medium (hydrogel). However, a tendency between ${P}_{\mathrm{dec}}$ and SoS estimation error ratios was observed even when considering the variation in results among wires.

## Discussion

The conducted experiments revealed that $\widehat{c}$ was overestimated when the probe was rotated by $\theta$ relative to the vessel short-axis plane [Fig. 1(2)], and that ${P}_{\mathrm{dec}}$ decreased. Furthermore, a similar relationship between the ${P}_{\mathrm{dec}}$ and the SoS estimation error ratio was observed for the water tank and the phantom experiments, although the acoustic properties of the propagation media and target cylinders were different between the experiments, as listed in Table 1.

Several studies have reported the measurement results of SoS in human liver [22] and rat liver [13, 23]. Ino et al. [23] also reported the measured value of attenuation in rat liver. The range of reported values differed among studies; however, the differences among reported values (SoS in liver: approximately 1510–1580 m/s [13, 22, 23], attenuation coefficient in liver: approximately 0.5 dB/cm/MHz or higher measured by 5 MHz transducer [23]) and acoustic properties (SoS and attenuation) of the phantom were smaller than the differences between the water tank and phantom experiments shown in Table 1. These results suggest that the relationship between the decrease in power, ${P}_{\mathrm{dec}}$, and the SoS estimation error ratio is independent of or less dependent on the acoustic properties of the propagation medium and target blood vessels, which are generally unknown in vivo.

As described in the Methods section, the relationship between the ${P}_{\mathrm{dec}}$ and the SoS estimation error ratio may depend on the ultrasonically transmitted beam conditions and depth of the target cylinder. However, this relationship can be preliminarily evaluated for each beam condition (machine setting) and target depth using experiments similar to those conducted in this study. In in vivo measurements, the actual running directions ($\theta$) of the blood vessels in the liver cannot be known. However, by preliminarily determining the threshold of ${P}_{\mathrm{dec}}$ for each beam condition (machine setting) and target depth, it is possible to determine whether the scattered waves from a blood vessel in the liver can be used to correctly estimate the SoS by evaluating ${P}_{\mathrm{dec}}$. For example, when the same beam conditions as in this study are used and blood vessels positioned at a depth of 30 mm are used for the SoS estimation target, we can determine that a 2% or larger SoS estimation error may occur if ${P}_{\mathrm{dec}}<-10$ dB.

Thus, the SoS in the liver can be stably estimated by applying the SoS estimation method in our previous studies [4, 15] only to vessels in which ${P}_{\mathrm{dec}}$ satisfies the desired SoS estimation accuracy. If this condition is satisfied, the SoS can be estimated using the method described in Ref. [4]. Subsequently, if the target can be considered as a point scatterer, the estimated SoS should be used as it is; if it has a non-negligible size, it should be corrected using the method described in Ref. [15]. In this study, a target cylinder with a small diameter (200 $\upmu$m for the water tank experiment and 80 $\upmu$m for the phantom) was examined to distinguish the effect of probe rotation angle $\theta$ from that of the scatterer size described in previous studies [14, 15]. The examination of more complicated conditions, where the probe rotates to blood vessels with a non-negligible diameter, will be the subject of our future study. The conditions in which the target cylinder does not have a straight shape should also be examined in future studies.

As discussed in the Results section, ${P}_{\mathrm{dec}}$ for $\theta =60^\circ$ was affected by weak scattered signals from the surrounding scattering sources in the phantom experiment. This effect may also be caused in in vivo measurements of the liver. However, this effect becomes a problem only for the low ${P}_{\mathrm{dec}}$ condition (that is, large $\theta$ condition causing a decrease in power around the edge of elements). The low ${P}_{\mathrm{dec}}$ condition (that is, large $\theta$ condition) causes large SoS estimation error, as shown in Fig. 5, and this is the condition that should be removed for accurate SoS estimation. Thus, the effect of weak scattered signals from surrounding tissues does not become a problem for this study.

In this study, we experimentally confirmed that the decrease in power, ${P}_{\mathrm{dec}}$, could be used to judge the target signal condition (probe rotation angle $\theta$) causing the SoS estimation error. However, the reason for the decrease in power owing to rotation angle $\theta$ must be theoretically elucidated in future studies. When the target has a cylindrical structure, the transmitted ultrasound waves are scattered at multiple positions on the cylinder surface, and the interfered waves are received by the elements in the probe. This interference may cause a decrease in power around the edge of the elements. In addition, this interference changes as a result of the geometric relationship between the target and the element array in the probe; therefore, the amount of decrease in power may be systematically changed by rotation angle $\theta$.

The liver contains many blood vessels [16, 17]; therefore, it is possible that signals from the neighboring blood vessels interfered with each other. In our experiments, the results for a single target (the water experiment) and those for several targets (the phantom experiment) showed a similar tendency; that is, the effect of this interference among the target wires was not observed in the phantom experiment. However, the effects of this interference should be examined in greater detail in future studies.

In this study, a linear array probe was used; however, application of the proposed method to a convex probe will expand its applicability in liver measurements. For this purpose, it is necessary to introduce the curvature factor of the convex probe into the proposed method, which should be examined in detail in future studies. Another choice is to use a linear array probe for SoS measurement in the liver, although applicable depth and field of view are restricted. Shear wave elastography has been widely studied to evaluate diffuse liver diseases although measurement position and range of the region of interest are restricted for accurate measurements [24]. Similarly, SoS measurement using a linear array probe may be applicable to diagnosis of diffuse liver diseases.

Several SoS estimation methods assume that the scattering source is a point scatterer. We demonstrated that the SoS is overestimated if the target size is non-negligible [14, 15]. Based on the results of this study, the running directions of vessels in addition to the target size must be carefully considered for applications utilizing absolute SoS values.

## Conclusion

In this study, the effect of probe rotation angle $\theta$ relative to the short-axis plane of the blood vessel on SoS estimation was examined through water tank and phantom experiments. When the probe was rotated by $\theta$ relative to the short-axis plane of the vessel, the SoS was misestimated, and a decrease in power at the edge elements relative to the center element was observed. Therefore, we proposed a method to distinguish the presence or the absence of SoS misestimation caused by the probe rotation without information on $\theta$ by thresholding the decrease in the power of the received signals at the edge elements relative to the center element. The proposed method can be used for in vivo SoS estimation by combining the methods described in Refs. [4] and [15]. In future, this method will be used to estimate the SoS in the liver.

## Data Availability

The data are available from the corresponding author upon reasonable request.
